# Medical and Physical Intelligence

**Published:** 1799-08

**Authors:** 


### MEDICAL AND PHYSICAL

### *INTELLIGENCE,*

### (Original and Selected.)

IN the 87th Number of the " Annales de Chimie," we met with a curious paper by Cit. WELTER, *on some particular matters discovered in animal substances, when treated by the nitric acid.* Cit. W. having made an experiment with the nitric acid on silk, with a view to expel the oxalic acid, was surprized at the end of the process, when he did not find the smallest particle of it, but obtained an unknown salt, of a soft nature, and the colour of gold, which, on being touched with a piece of burning charcoal, exploded like gunpowder. The following is an account of the whole process.

On one part of silk, he poured six parts of the common nitric acid, to which he added a small quantity of concentrated nitric acid. The mixture was left standing for two days, and then distilled. He mixed what had passed into the receiver with what remained in the retort, and put the whole on a filter. The oxalic acid crystallized sooner than he wished, and he again put the whole into the retort, together with the water which had been used to cleanse the filter. He made one part of the water pass over by distillation, and attempted to crystalize the residuum, but did not succeed.

He then put the residuum into the retort, with what was left in the receiver, and distilled it again. At length, having several times repeated the same series of operations, he obtained an acid liquor, containing small granulated crystals, and reduced nearly to the weight of the silk employed.

This liquor, when submitted to different experiments, did not afford the smallest trace of the oxalic acid; it was yellow, and left a stain of that colour upon the fingers: it communicated to white silk a beautiful yellow, which was not affected by washing it in water.

To saturate this liquor, he mixed some chalk with it, and finished the saturation by the addition of lime; on examining it again, he poured on it some alcohol. An apparently gummy matter was separated from it, which was kept separate. The alcohol was spread over the water and evaporated. A yellow substance remained, with the solutions of nitrat and muriat of lime. Having at length decomposed these salts by the carbonate of potash and separated the carbonate of lime, the filtered liquor was emptied into a receiver, and placed in a sand-bath. The next day he fouud gilt crystals adhering to the sides of the receiver as fine as silk, which detonated like gunpowder, and which, if properly managed, would have produced the report of a musket. The smoke of this explosion resembled that of burnt resin.

This gilt salt was soluble in water and alcohol; and it crystallized in a cold temperature.

The oxygenated muriatic acid, poured on the solution, destroyed the yellow colour, and rendered it of a milky appearance.

The sulphuric acid being poured on the crystals, afforded the smell of nitric acid. The muriatic acid occasioned a precipitate of small whitish and micacious crystals, volatile in fire, and emitting a smoke of a bitter taste. This smoke was inflammable, and burnt like the essential oils.

He made experiments on new silk with the nitric acid, and after having obtained obtained by different trials, some crystals of the oxalic acid, he poured some of the weak nitric acid upon the residuum, which thickened to the consistence of honey; and after having somewhat heated the mixture, to dissolve the whole of it, he let it rest for two days. He then found more of the oxalic acid crystals, and some other granulated crystals of a yellowish colour, extremely bitter, and without the least acidity, which dissolved the saliva, and left yellow spots on the tongue. They were volatilized by fire, and not affected by the concentrated nitric acid, which only discoloured them; but plunging them in water was sufficient to make the colour return.

After having saturated with potass a small portion of the nitric acid, combined with this substance, he evaporated the mixture, and found that the residuum took fire like the silky salt before-mentioned. Hence the author imagined, that this last compound was a triple salt, composed of nitrat of potass, combined with the yellow substance, which he called *animal bitter*.

The crystals of this yellow bitter, when viewed sideways, appeared like octaeders, the two opposite ends of which were truncated; there then remained square rectangular plates, the edges of which were cut by a plane on two sides. As animal substances become yellow by bringing them in contact with the nitric acid, he endeavoured to draw the bitter from raw beef, but found it combined with another substance, unchangeable, like the former, by the nitric acid. This combination was soluble in the concentrated nitric acid, and was separated by water in the form of a yellow powder, which retained its colour when exposed to the air, and might perhaps be useful for painting.

What led him to presume that the yellow powder alluded to was composed of the bitter and another new substance was, that by dipping a sponge in the nitric acid, he obtained this latter substance, which was colourless, soluble in the concentrated nitric acid, the same as the preceding powder and was precipitated by water like the former; he also found that the bitter was particularly eager to combine with animal substances, and by what means its colours were fixed.

The author here observes, that each of the experiments were only made once; and not knowing to what particular circumstances of his pursuit he should attribute these new results, he preferred detailing the whole.

He concludes with remarking, that the bile, perhaps, owes its colour and bitterness to what he calls *animal bitter*.

---

Cit. Brugnatelli, in a letter to Van Mons, inserted in the 87th Number of the 'Annales de Chimie,' informs us that he has lately obtained a great quantity of a *peculiar resin, by distilling the nitric acid with indigo.* The solution of this new substance in alcohol has a deep yellow colour, which stains the skin and nails, and cannot be discharged by water. Brugnatelli calls this substance the indigoferous resin; and adds that it may be useful in some of the arts, especially as about one half of the indigo may thus be converted into resin.

---

In another part of his letter Brugnatelli states, that he has caused the mercury in the thermometer to sink to thirty degrees below the freezing point, by a mixture of the muriat of lime with snow. If the cold should become more intense, he hopes to be enabled to apply this artificial cold to the most important purposes.

---

On the *inflammation of Ethers by means of Acids*, the same author communicates the following experiment:—Having poured upon two ounces of the the pureft alcohol an equal quantity of the nitric acid, and added, two minutes after, the fame proportion of the common fulphuric acid, at two different times, there firft arofe from this mixture a white vapour, which was fucceeded by a very lively ignition, fimilar to that which takes place after pouring nitric acid on fpirits of turpentine. At the fame time a red vapour was evolved with nitrous gas, and the copious fumes were condenfed by the cold of the atmofphere. Thefe fumes filled the laboratory with an odour not unlike that arifing from ether.

He repeated this experiment in clofe veffels; and, in order to fucceed more completely, he introduced into a tubulated retort, two ounces of the pureft alcohol, and the fame quantity of the nitric acid, having adapted to the retort two receivers with long orifices. After clofing the apertures, he covered the receivers with cloths dipped in cold water, the temperature of which was only one degree above zero. He then introduced the fulphuric acid into the retort, conftructed fo that not the fmalleft bubble of gas could efcape. The effervefcence and combuftion were very great; after which the firft receiver contained a portion of the original mixture, which had been expelled from the retort by the violent effervefcence: in the fecond he found the nitrous ether, which was flightly acid; and on the furface of this liquor a greenifh oil was formed, which emitted a very agreeable aromatic odour.

On pouring the fulphuric acid upon nitrous ether, an effervefcence took place, fimilar to that which arifes from the decompofition of an alkaline carbonat, by means of a mineral acid. The ether obtained from fulphuric acid, when employed inftead of the alkohol ufed in the firft experiment, produced a violent combuftion. Brugnatelli intends to continue thefe experiments, and to communicate the refult of them to the public.

---

In the 87th Number of the "Annales de Chimie" are inferted fome obfervations on the muriatic acid, being the refult of various experiments made by Cit. Tassaert, with a view to effect the decompofition of that fubftance.

The author was induced to undertake thefe experiments, by reading a quotation from a work publifhed by M. Girtanner, in which he remarks, "that the muriatic acid abounds in the mineral kingdom; in fea-water it is found combined with lime, magnefia, and foda, as well as rock-falt, and with foda alone. Hitherto the conftituent parts of this acid were unknown, and it is only from analogy we conjecture that oxygen forms a part in its compofition; but I have at length fucceeded in decompofing it into hydrogen and oxygen." From a feries of eighteen experiments, which Girtanner made with different fubftances, to effect the decompofition of the acid, he gives the following refult, namely, "that water bears the fame relation to the muriatic acid which air does to the nitric acid; and it may be readily conceived why the former is fo abundantly contained in fea-water. The muriatic acid cannot exift, otherwife than in a gafeous ftate, in the temperature, and under the preffure of atmofpheric air: and in order to condenfe it, we are obliged to employ water with which it readily combines."

Taffaert afferts, that Girtanner has too haftily formed thefe conclufions, and that he has thus been led into error; for he firft obferved that he had obtained the muriatic acid deprived of water, and at the end of his experiments he affures us, that this acid can exift only in a gafeous ftate, unlefs it be combined with water.

After detailing feveral experiments made by him in the laboratory of the Mineral School, Taffaert endeavours to refute fome of thofe made by Girtanner,

Girtanner, and concludes with the following remark, " that we ought ſtill to conſider the muriatic acid as a ſimple body, as we have not yet diſcovered the means of decompoſing it."

---

Dr. Carradori ſuppoſes it no longer doubtful, that nocturnal birds of prey digeſt vegetable ſubſtances. It appears from his experiments, that ſuch birds may be kept alive and nouriſhed on vegetable food, although they ſeem to have a natural averſion to it; hence Carradori endeavours to explode the erroneous opinion, that the gaſtric juice of theſe birds was homogeneous only with animal ſubſtances.

This aſſertion, as well as the experiments of Carradori, that carnivorous animals receive nouriſhment from plants, derives additional evidence from the diſcovery made by Fourcroy, relative to the exiſtence of gluten, albumen, and jelly, in vegetable bodies.—See the " *Annales de Chimie,*" No. 86. p. 171.

---

The ſame author communicates ſome new experiments and obſervations on the reſpiration of frogs and fiſh, from which he concludes, that aquatic frogs are under the neceſſity of reſpiring, in order to preſerve their life. He obſerves, that theſe animals, if kept under water, lived much longer when the veſſels containing them were left open, than when they were ſhut; and that their exiſtence was long or ſhort, in proportion to the extent of the water in which they were taken. If the water was covered with a thin ſurface of oil, they lived but a ſhort time; and when placed in pure oil, they died in forty minutes.

The author alſo wiſhed to aſcertain, how far water is neceſſary to ſupport the life of theſe animals. He remarks that frogs, immerſed to the neck in water, died in one third leſs time than thoſe which were kept dry.

When fiſh were incloſed in the receiver, which was in part filled with air, it was obſerved, that they did not conſume any of this elaſtic fluid. *Ibid.* No. 86.

---

As Bergman, and moſt other chemiſts, have conſidered *the oxalic acid as a re-agent on lime*, Brugnatelli has endeavoured to ſhew the error of that opinion, by ſeveral experiments, in which the preſence of lime has been aſcertained by all other known re-agents, the oxalic acid excepted.

In a mixture of lime-water with a ſolution of the muriat of barytes, the oxalic acid did not produce the leaſt precipitation; but lime-water yielded a precipitate on mixing this ſolution with the oxalic acid:—the ſame reſult was obſerved with the nitrat of barytes.

The acidulated phoſphat of lime, obtained by the decompoſition of bones in the ſulphuric acid, readily imparted its baſe to the oxalic acid; but upon adding a ſmall quantity of ſulphuric acid to this acidulated ſalt, a portion of lime was precipitated in the form of ſulphat, and the remainder of this earth had not the leaſt attraction for the oxalic acid: notwithſtanding which, the potaſs and ammoniac afforded an abundant precipitate.

The nitrat of lime was precipitated by the oxalic acid; but this acidulated nitrat, or even with an exceſs of the acid, did not undergo any change.—The acidulated muriat of lime, as well as the acidulated tartarit of lime, gave ſimilar reſults; and the ſame took place with the acidulated ſulphat of lime, although the alkalies ſeparated the lime from all theſe ſalts.

Several other acidulated ſalts of the ſame earth were not in the leaſt acted upon by the oxalic acid. *Ibid.*

---

Dr.

Dr. Carradori, in his 15th volume of the "*Annali di Chimica*," gives a deſcription of a very convenient apparatus for impregnating liquids with the carbonic acid ; and we have only to regret that he has not furniſhed us with a more perfect and ſatisfactory account:—This apparatus conſiſts of a caſk bound with iron hoops, and large enough to contain about four pailfuls; no more than two thirds of that veſſel ſhould be filled, and a communication eſtabliſhed between it and a glaſs bottle, which contains powdered marble and ſulphuric acid. An aperture ſhould be made in the upper end of the caſk, into which a curved tube is introduced, to carry off the ſuperabundant gas. The author aſſures us, that with this apparatus a perſon may in leſs than half an hour, ſaturate any quantity of water in proportion to the carbonic acid diſengaged.

Van Mons, who has extracted this article from the aforementioned work of Brugnatelli, obſerves on this occaſion, that ſeveral other methods have been lately propoſed for the ſaturation of water with alkalies, and the carbonic acid; but that an eſſential improvement might be made on the apparatus propoſed by the indefatigable chemiſt of Pavia, namely, by conveying the decompoſing acid on the carbonate of lime, by a ſlow inſtillation; which might be effected by paſſing the communicating tube tranſverſely over a funnel adjuſted to the mouth of the receiver. By this contrivance, a regular and gradual diſengagement of the gas will take place, none of it will be loſt, and the acceſs of atmoſpheric air prevented ; which, by mixing with the gas diſengaged, neceſſarily retards the ſaturation.

It is worthy of remark, that the difficulty of procuring, in Italy, glaſs veſſels provided with more than one neck, firſt induced the author to adopt the apparatus here deſcribed. The ſaturation may be greatly facilitated by employing a ſimple gaſometer in conducting this proceſs. *Ibid.*

---

In a Memoir of Descemet, *on the irritability inherent in the ſtamina of the flowers of the ſorrel-thorn* (*l'epine-vinette*), we meet with the following remark, " that this irritability by which the ſtamina, on being ſlightly touched, incline nearly two inches, appears to be deſtined by Nature for the purpoſe of promoting the act of generation. *Ibid.*

---

Pierre Smith has, in the 94th volume of the Literary Journal of Naples, communicated ſome experiments which lead to the concluſion, that the muſcular parts of living animals, when wounded or ſtimulated, have the faculty of ſeparating *a humour ſimilar in its effects to the gaſtric juice*, and acting on animal and vegetable ſubſtances in the ſame manner as this juice does on the aliment taken into the ſtomach.

The author imagines, that the chyle is a ſolution of vegetable and animal ſubſtances in the gaſtric juice; and that the pus is a ſpecies of chyle, compoſed of the ſame juice and animal ſubſtance in a ſphacelated ſtate. His proofs in ſupport of theſe aſſertions, are uniformly founded on the property which this ſub-cutaneous humour poſſeſſes, of coagulating milk and diſſolving animal and vegetable ſubſtances. Dr. Carradori however is of opinion, that theſe proofs, being deduced from properties common to a variety of bodies, and particularly ſweat, are not ſatisfactory: and he rationally remarks on the ſuppoſition, if the juice alluded to poſſeſs all the properties attributed to it by M. Smith, that animals ought to derive nouriſhment from the introduction of alimentary ſubſtances under the ſkin.—" I have preſerved," ſays he, " for ſeveral days, the life of a robuſt man (who, in conſequence of a wound in the throat, could not take any nouriſhment), by cauſing causing different parts of his body to be rubbed with a sponge dipped alternately in wine and strong broth." *Ibid.*

---

M. Von Mussin-Puschkin has requested M. Von. Crell, the editor of the German "Annals of Chemistry" to make some experiments on what he called the combination of the muriatic with the sulphuric acid; from which he promised himself very interesting results. The French editors of the "Annales de Chimie" remark, on this subject, that they have formerly complied with a similar request of the learned President of Petersburg, and found that the two acids did not manifest any new properties by their union; but, when the mixture was made with the acids in a concentrated state, the sulphuric acid, on account of its uncommon attraction for water, displaced and expelled the muriatic gas which was condensed by its dissolution in this liquid. When equal parts of the two acids were used, and the mixture was made with the whole mixture at once, the effervescence became so strong, and the disengagement of gas so considerable and instantaneous, that the liquor overflowed the highest vessels, and not the smallest trace of the muriatic acid could be discovered in the mixture. A small quantity of caloric only was disengaged during this process, by which it is manifest, that the base of the muriatic gas consumes, in a great measure, that which is disengaged from the water, by the sulphuric acid. *Ibid.* No. 88.

---

Professor Lowitz thought it practicable to reduce, by partial and gradual disoxygenation, such vegetable acids as contain much oxygen, to the state of those which are less oxygenated: he hoped, by the same means, to convert the acetous acid into the oxalic, and the latter into the tartarous acid, &c. With this intention, he boiled the acetous acid concentrated by cold, with phosphorus, but did not succeed in the experiment.

M. Mussin made similar experiments, but with no better success. It appeared that the acids parted only with a portion of their oxygen to phosphorus, in order to re-absorb it from the atmosphere. This obstacle, however, it would not be very difficult to surmount, but that disoxygenation could not be attended with the transition of the vegetable acid through its different modifications hitherto known: for the particular states of its composition do not depend less on the different proportions of its component elementary parts, than on those of its oxygen. Hence the acetous acid, by the addition of a new portion of oxygen, or by the action of the concentrated sulphuric acid, undergoes a change in the proportion of its elementary principles, hydrogen and carbon; and it is not changed into a more oxygenated acid, but into a new acid, which ought to be distinguished, in the nomenclature, by a particular name. *Ibid.*

---

Kasteleyn has lately observed that, in a solution made of the muriat of potass and the carbonat of soda, one or the other of these salts may be crystallized at pleasure, according to the temperature in which the lixivium is kept. Below 15° of Réaumur, the muriat forms crystals; above that temperature, the carbonat crystallizes.

The author remarks, that he has already shown, that the acids of these two salts change their bases in different temperatures. *Ibid.* No. 89.

---

M. Hulsenkamp has lately published a Latin dissertation on ether, obtained from the sulphuric acid; of which the following is an extract:

Professor Driessen, of Groninguen, having made experiments on ether, by treating it with the nitric acid, to discover the presence of the sulphuric acid, after the manner of Scheele, obtained a considerable quantity of crystals of the oxalic acid. Hence he concluded, that the precipitate which the Swedish chemist found formed in this ether, by means of the nitrat of barytes, was not the sulphat, but the oxalite of that earth. This observation confirms what he (Hulsenkamp) had formerly remarked on the possibility of converting the whole of a given quantity of alcohol into oxalic oxyd by distilling this liquor several times over with the nitric acid, though the oxyd was mixed with a small quantity of nitric and acetous acid.

The French editor observes, that if Scheele is mistaken in his method of demonstrating the presence of the sulphuric acid, or rather of sulphur imperfectly oxygenated in ether, M. Hulsenkamp has likewise been deceived in his turn, and is far less excusable, as he has mistaken the precipitate, formed during the inflammation of a mixture of ether, alcohol, and a solution of the nitrat of barytes, for the real sulphat of barytes. This precipitate should rather have been called the carbonat of that earth, formed from the nitrat of barytes, from which the acid is decomposed into its principles, while it yields its oxygen to the hydrogen and carbon of the ether and the alcohol. The acid which resulted from the oxygenation of the carbon of these liquids, by uniting with the precipitated barytes, rendered this substance insoluble in water.

---

In an extract from a Latin dissertation on various chemical and pharmaceutical preparations, by M. Tiebol, we find the following experiment worthy of notice:—The author precipitated, with the muriat of mercury, the whole quantity of that metal contained in a solution made by the nitric acid. Three parts of mercury, and one and a half parts of pure aquafortis, were gradually heated to the boiling point, in a tubulated retort, on the top of which he had fixed a thermometer. After two hours and a half, twenty parts of water were added to this solution, which was then precipitated with a lixivium made of the muriat of soda. M. Tiebol examined the supernatant liquid, by the test of the ammonia, and did not obtain any precipitate. The muriat, however, which was formed in this process, weighed 0.06 less than the mercury employed.

---

Kasteleyn has also made the curious observation, that the martial flowers of ammoniac, though very pale, when exposed to the rays of the sun, acquire a deep orange colour, which they lose again in the shade. Enclosed in a phial, after they have attracted the rays of light, these flowers retain all the vivacity of colour before acquired: the effect in these cases appears to be produced by the alternate disoxydation and re-oxydation of the iron. *Ibid.*

---

M. Wurser gives an account of the economical employment of nitric acid in Pickel's manufactory at Würzburg; where the manner of re-oxygenating this acid, when decomposed by copper, attracted the whole of his attention. The nitrous gas disengaged in the solution of copper is introduced into receivers containing water and filings of that metal. This gas, by its contact with atmospheric air, is re-oxygenated, dissolved by the water, and again decomposed by the copper. *Ibid.*

---

The same chemist makes the following remark on the preparation of *Hahneman's soluble mercury*. "It is immaterial whether the mercury is precipitated by the ammoniac, or by either the vegetable or mineral alkali, or even by lime. The result, after washing and edulcorating the solution is uniformly an an oxyd of a dark-brown colour, or mercury in an almost reduced state. The great point to be attended to, is, to have a certain criterion for the comparison of colour. As this mercurial preparation is much used in Germany, Kasteleyn advises the operator, not to expose mercurial preparations to the disoxygenating influence of light. *Ibid.*

---

LEONHARDI, the German translator of Macquer's Chemical Dictionary, has lately published an essay "*On the reconciliation between the theories of phlogiston and oxygen.*" VAN MONS remarks on this occasion "that this is a puerile attempt at a mixed theory, behind which the German chemists have entrenched themselves after their defeat.*" *Ibid.*

---

Prof. BERGMAN, of Leyden, has lately discovered *a test for ascertaining whether cotton be adulterated with an admixture of wool*; by submitting it to the action of oxygenated muriatic acid, which bleaches the cotton, while it imparts a yellow tinge to the wool. By similar means he has been enabled to distinguish, with accuracy, the *medullary substance* of the brain from that of the *nerves*, and to trace the latter even to their most hidden origins. *Ibid.*

---

As the solution of *caoutchouc*, or elastic gum, is an object equally deserving the attention of the chemist and the medical practitioner, we shall extract a few passages relative to this subject, from SAINT FOND'S "*Travels into England and Scotland, &c.*" of which an English translation has lately been published.—The author, in a conversation with CAVALLO, applied to that learned philosopher for an explanation respecting an article inserted in Macquer's Chemical Dictionary, which has given occasion to several strictures that appeared on the assertions of that celebrated chemist, relative to the method of dissolving elastic gum in ether.

It is certain, said Saint Fond, that vitriolic ether, as it is usually prepared, does not dissolve elastic gum. But on the death of Macquer, of whose chemical cabinet I became the purchaser, I found three small decanters, in one of which there was elastic gum, perfectly dissolved in ether. The other two decanters contained some likewise, which appeared to be partly dissolved; but it was precipitated to the bottom, in a state a little thicker than turpentine, and was found incapable of mixing with the ether in the bottle. That which contained the elastic gum in a state of perfect solution, had a lable with this inscription, in the hand writing of Macquer: *Elastic gum dissolved in ether, sent from London.*" I mentioned this, to be informed whether you know any one in London who has employed ether in dissolving caoutchouc, and what were the ingredients used in addition to it, or the preparation which it received.

"You could not have addressed yourself," replied Cavallo, "to one who is able procure a more complete answer to your questions than myself. We shall go this morning to visit the workshops of some artists; and as the person who discovered the process for dissolving elastic gum is in our way, we shall give him a call; hence your wishes shall be very soon gratified."

I accepted his offer, and in about an hour afterwards we went to the house of Mr. WINCH, an apothecary who received us politely, and told me that he was the person who had addressed to Macquer at Paris, a bottle of elastic gum well dissolved in ether, and that in a letter to the French Chemist, he assured him

---

* We humbly advise Cit. Van Mons to make himself better acquainted with the latest chemical writings of GREN, RICHTER, GOETTLING HERMBSTADT in the original, and he will, we doubt not, there learn, that this *defeat* is not greater than that of one hypothesis may boast over another. W.

him that the ether did not contain the smallest mixture. Macquer, who found the elastic gum in perfect union with ether, of which the transparency was not in the least altered, and who, on examining the ether, found it totally free from any extraneous substance, sincerely believed that pure ether was the real solvent; and notwithstanding his succeeding but imperfectly himself, though he employed the best ether, he probably persuaded himself that what he used was still insufficiently rectified.

"I did not, indeed," said Mr. Winch, "send him an account of the process which I used, but it is nevertheless true, that the ether is unmixed, and that the whole depends on a very simple preparation.

"Cavallo, who is the friend of Mr. Winch, said, that he intended to perform the experiment the next day, at his own house, and that I should be a witness of it. It consists in the following process:—A pound of good vitriolic ether is taken, and put into a bottle, capable of containing about four pounds of any common liquid. On this ether are poured two pounds of pure water, the bottle is then stopped, held with the mouth downwards, and strongly shaken, in order to mix the two liquors. On discontinuing the shaking, the ether soon swims uppermost; the bottle is still held in the same position, and cautiously opened, keeping the thumb on the mouth of it. The water is by this means easily let off, and collected in a vessel below. The same operation is repeated two or three times, with new quantities of water, until the sixteen ounces of ether are reduced to about five ounces. It is this purified remainder that is found to be the most perfect solvent of elastic gum, which is thrown into the ether, after being cut into small pieces. It begins to swell in a very short time; the ether penetrates it, and appears to act slowly on it at first; but at the end of five hours, or longer, the liquor is saturated, and remains transparent. If there be a surplus of elastic gum, it subsides to the bottom, which on being taken out of the bottle, may be moulded into any form, and will preserve its elasticity.

"To shew how the part which is completely dissolved is to be applied to to use, I shall describe the method employed by Cavallo, to form a tube of elastic gum.

"A small cylinder of pipe clay is first prepared, of the diameter and length of the intended tube. It is not necessary to bake it, but simply to let it dry.

"The ether, saturated with gum, is poured into a case of glass, or tin, which should be a little longer than the clay cylinder; this is filled up to the brim.

"The operator then plunges the whole leng h of the clay pipe into the ether, withdraws it suddenly, lets it remain for an instant in the air, re-plunges it anew, and repeats the operation in proportion to the intended thickness of the tube; for each immersion and evaporation produces a small coating.

"This being done, the clay cylinder, covered with elastic gum, is plunged into a vessel of water; the mould of clay is there speedily dissolved, and the gum remains in the state of a perfect tube.

"This method of dissolving and using elastic gum is ingenious, and in one respect resembles that employed by the natives of America, who form all their works in elastic gum, on moulds of clay. It may be objected, that the process with ether is too expensive. The objection holds good with re-respect to ordinary purposes; but the elastic gum has been so usefully employed in surgery and some other arts, that there are circumstances in which expence ought to be of no consideration. The process for making ether is besides so simplified, that it is not half so dear as it was formerly.

I should

I ſhould not forget to mention, that the water uſed in purifying the ether ought to be preſerved, becauſe a part of the ether mixed with it may be recovered by diſtillation."

---

Good Bougies and Catheters have long been a deſideratum, in the treatment of ſtrictures in the urethra, and for evacuating the urinary bladder.

W. Smyth, of Taviſtock-ſtreet, Covent Garden, has lately diſcovered a metallic compoſition, which unites the flexible property of lead to the white luſtre of ſilver. From this excellent compoſition, Mr. Smyth manufactures his "*Metallic Bougies, ſolid and hollow, as well as his flexible metallic catheters, for males and females.*" He recommends them with a degree of modeſty, deſerving much praiſe, *for the cure of ſtrictures only;* while he very properly remarks, that a *temperate regimen* ought to be obſerved during their uſe. For want of room we refer the reader to the printed directions given by the inventor.

---

We underſtand from a Correſpondent, that there is now preparing for the preſs, at Edinburgh, a work entitled, "*A free and impartial inquiry into the preſent ſtate of medical knowledge; together with a comparative view of the ſyſtems of Cullen, Brown, and Darwin, comprehendiug a body of information reſpecting the riſe and progreſs of the healing art, from its earlieſt dawn, down to the preſent time:*"—By Alexander Campbell, Eſq. author of "An Introduction to the Hiſtory of Poetry in Scotland," &c.

---

We have not been deficient in inquiries among our friends, reſpecting the ſucceſs, or failure, of the *Metallic Tractors*, but have not hitherto been ſo fortunate, as to obtain any ſatisfactory account on either ſide. We ſhall therefore be much obliged to any of our Correſpondents, who will favour us with authentic information on the ſubject.

---

In the "*Journal der Erfindungen,*" No. 6, we find a ſhort account of the extraordinary effects of the *Extractum Taxi*, which has lately been introduced into practice by ſeveral phyſicians in France. The beſt account of this remedy, as well as an accurate chemical analyſis of it, and ſeveral remarkable facts eſtabliſhing its medicinal virtues, have been given by Dr. Loder, in his Inaugural Diſſertation, entitled "*De Taxo baccata, Linn.*" 4to. 1794.—According to this writer, the extractum taxi is a narcotic remedy, and ſhould be given, in doſes from one or two grains, diſſolved either in water or ſpirits. The doſe may be gradually increaſed, whence this powerful remedy has proved uncommonly efficacious in curing obſtinate tertians (after the *material cauſe* was removed, ſays the humoral pathologiſt) rheumatiſms, epilepſy; and amenorrhœa. All theſe aſſertions are corroborated by hiſtories of caſes, and it is to be hoped that repeated experiments will confirm the efficacy of this new remedy.

